# Phytochemical Analysis, Antibacterial, and Antitumor Potential of *Hibiscus rosa-sinensis* Linn

**DOI:** 10.1155/sci5/2722306

**Published:** 2025-04-28

**Authors:** Neelma Munir, Sheza Ayaz Khilji, Sajal Rasool, Anam Khalil, Zahoor Ahmad Sajid

**Affiliations:** ^1^Department of Biotechnology, Lahore College for Women University, Lahore, Pakistan; ^2^Department of Botany, Division of Science and Technology, University of Education, Township Campus, Lahore, Pakistan; ^3^Plant Developmental and Regenerative Biology Laboratory, Institute of Botany, University of the Punjab, Lahore, Pakistan

**Keywords:** antimicrobial, antitumor potential, *Hibiscus rosa-sinensis*, phytochemicals

## Abstract

This study aimed to investigate the phytochemical composition, antibacterial activity, and antitumor potential of *Hibiscus rosa-sinensis* Linn, a widely used medicinal plant. Qualitative systematic phytochemical analysis was conducted on flower of *Hibiscus rosa-sinensis* for determining their antimicrobial and antitumor potential by using disc diffusion method and potato disc bioassay, respectively. Phytochemicals found in chloroform extract of *H. rosa-sinensis* were alkaloids, betacyanin's, tannins, and quinones in nature while in ethanol extract only alkaloids and betacyanin's were present. The findings demonstrated potent inhibitory effects of the extracts against both Gram-positive and Gram-negative bacteria. Chloroform extract exhibited more significant antibacterial activity as compared to ethanolic extract against various drug resistant pathogens such as *Salmonella typhi*, *Escherichia coli, Staphylococcus aureus, Klebsiella* sp.*, Pseudomonas aeruginosa*, and *Bacillus subtilis.* Furthermore, the antitumor activity of the *H. rosa-sinensis* extract was evaluated using in vitro assays against selected cancer cell-lines. These extracts exhibited significant antiproliferative effects such as ethanolic extract had more significant antitumor activity as compared to chloroform. After 24 h zones of inhibition were analyzed and measured using ruler. The diameter of zones of inhibition of chloroform flower extract against *E. coli, S. aureus, Klebsiella* sp.*, Pseudomonas aeruginosa*, and *B. subtilis, S. typhi* was 11, 8, 13, 17, 10, and 23 mm, while ethanolic extract had 6, 0, 6, 10, 7, and 10 mm, zone of inhibition, respectively. Flower extracts by inhibiting the formation of crown gall tumors suggesting their potential as sources of natural anticancer compounds. These results provided valuable insights into the phytochemical composition and therapeutic potential of *H. rosa-sinensis*. Additional investigations into the bioactive components of *H. rosa-sinensis* might result in the creation of new antibacterial and anticancer medicines for use in a range of medical applications.

## 1. Introduction

In ancient times, herbal medicines were widely used for a variety of health concerns by diverse populations. A wide range of plants is used in traditional medicinal practices such as Unani Medicine, Chinese Traditional Medicine, Ayurvedic Medicine [1]. Herbal medicines are known for their potential in various treatments such as cancer by exhibiting anticancer properties emphasizing their role as complementary treatments [2]. Plant based medicines offer a promising alternative to traditional antibiotics in terms of efficacy and safety. Studies have shown that herbal preparations can reduce the frequency of prescribing systemic antibiotics in patients with acute respiratory infections, leading to a decrease in the duration of temporary disability [3]. Additionally, herbal drugs have been found to be generally safe for treating uncomplicated infections of the urinary or respiratory tract, preserving the microbiota and avoiding the negative impacts associated with antibiotic use [4, 5]. The use of herbal medicines presents a valuable opportunity to address infections while minimizing the risks associated with antibiotic resistance and microbiota disruption. While the phytochemicals present in medicinal plants provide antioxidant, antibacterial, and antipyretic effects, contributing to their therapeutic properties and making them nontoxic treatment options for various ailments [6]. After exploring their potential and effective properties, the demand for traditional herbal medicines has surged globally, especially in economically disadvantaged regions, for treating various diseases and for cosmetic and nutraceutical purposes.

*Hibiscus rosa-sinensis*, commonly known as China rose, possesses a wide array of medicinal properties as documented in various research papers. The plant is rich in bioactive compounds such as flavonoids, tannins, terpenoids, saponins, and alkaloids, contributing to its therapeutic effects [7]. These properties enable it to exhibit hypotensive, antipyretic, anti-inflammatory, anticancer, antioxidant, antibacterial, antidiabetic, wound healing, and abortifacient activities [8]. Additionally, the plant's leaves, barks, roots, and flowers have been traditionally used to treat various ailments due to its antioxidant, antimicrobial, antidiabetic, antiulcer, hepatoprotective, and anti-inflammatory qualities [9].

Phytochemicals are bioactive compounds found in plants, playing crucial roles in plant defense mechanisms, growth regulation, and attracting pollinators [10]. Phytochemicals are not essential for plant survival but are vital for human health, offering protection against chronic diseases like heart disease, diabetes, and cancer [11]. They exhibit biological activities such as cytotoxicity, antioxidant properties, and interference with cellular pathways, making them valuable in medicine, food, and cosmetics industries [12]. The exploration of phytochemicals aligns with the idea of utilizing nature's gifts for human well-being, emphasizing the importance of harnessing these natural compounds for their curative potential.

Previous studies on phytochemicals have highlighted their potential in various fields such as cancer treatment, antimicrobial activities, and neuroprotection. Research has shown that compounds like erypoegin K from *Erythrina poeppigiana* exhibit potent antitumor effects through apoptosis induction and topoisomerase II inhibition [13]. Additionally, phytochemicals have been explored for their antimicrobial properties, with extracts from plants like *Rosmarinus officinalis* and *Zingiber officinale* showing activity against bacterial and fungal species [14]. Plants have phytochemicals that can be used to treat various diseases showcasing the potential of phytochemicals in combating drug-resistant infections [15, 16].

*Salmonella typhi*, *Escherichia coli*, *Staphylococcus aureus*, *K. pneumoniae*, *P. aeruginosa*, and *Bacillus subtilis* are significant for microbial infections discussed in the provided research contexts. Studies have highlighted the global spread of multidrug-resistant strains of *Salmonella* spp., *E. coli*, and *S*. *aureus*, emphasizing the need for epidemiological surveys and antimicrobial development [17]. Various antibacterial agents, including plant-derived extracts and synthetic compounds like Sulfamethoxazole, have been evaluated for their efficacy against these pathogens with some showing promising results in inhibiting bacterial growth [18, 19]. Additionally, research on the antibacterial activity of ethanolic extracts from avocado leaves against *E. coli, S*. *typhi,* and *P*. *aeruginosa* has demonstrated potential alternative treatments for these Gram-negative bacteria [20].

Diabetic foot ulcers (DFU) are a significant complication of diabetes mellitus, often leading to infections and potential amputation [21]. Studies have shown that DFU infections are typically polymicrobial with common pathogens including *S*. *aureus*, *P*. *aeruginosa*, *E*. *coli*, and *Proteus* species [22]. The prevalence of multidrug resistant organisms in DFU patients is alarmingly high, particularly in developing countries like India and Pakistan [21, 23]. Given the high prevalence of antibiotic resistance, it is crucial to identify causative organisms and their antibiotic sensitivity profiles to guide appropriate treatment and improve outcomes in DFU patients.

The disc diffusion assay is a widely used method for assessing antimicrobial activity due to its simplicity, low cost and ability to screen multiple samples [24]. This technique is particularly useful for evaluating algal extracts and antibiotic pretreatment in epidemiological studies [24, 25].

*Agrobacterium tumefaciens* infection on potato discs has been studied as a model system for crown gall tumorigenesis. [26]. The infection process involves specific bacterial attachment to the plant surface within 10 min, which is necessary for tumor formation [27]. The potato disc tumor induction assay using *A. tumefaciens* has been widely used to screen for antitumor activity. This assay effectively detects antineoplastic activity regardless of the drug's mode of action [28]. It serves as a safe, simple, and cost-effective prescreen for antitumor activity, showing better predictive power than cytotoxicity assays [29].

All the content cited in above paragraphs show the importance of medicinal plants for human beings and animals. Medicinal plants are considered as a rich source of ingredients which can be used in drug development either pharmacopoeiol, nonpharmacopoeiol, or synthetic drugs. So, it would be significant to examine the roles of phytochemicals. The aim of this study is to done phytochemical analysis of chloroform and ethanol extract of flowers of *Hibiscus rosa-sinensis* and to determine their antimicrobial and antitumor potential to fulfill the research gap.

## 2. Materials and Methods

### 2.1. Collection and Sterilization of Plant Material

Flowers (0.5 kg) of *Hibiscus rosa-sinensis* Linn (Voucher No. Bot-273) were collected (March-April) from mature plants grown at University of the Punjab Lahore, Pakistan (31° 30′ 15″ North, 74° 18′ 23″ East). The flowers were washed under running tap water and sterilized with 15% NaOCl for 15 min with gentle agitation then rinsed with distilled water to remove dust particles and microbes. Flowers were dried with blotting paper and grinded into fine powdered form to apply further steps of protocol [30].

### 2.2. Procurement of Bacterial Strains

Five different bacterial strains *Escherichia coli, Staphylococcus aureus, Klebsiella* sp.*, Pseudomonas aeruginosa and Salmonella typhi.*

*Bacillus subtilis isolated from foot* were procured from Biotechnology Laboratory, Lahore College for Women University, Lahore.

#### 2.2.1. Preparation of Nutrient Agar Plates

For preparing nutrient agar medium, 2.8 g agar was dissolved in 100 mL of d·H_2_O then autoclaved. In one petri-plate, about 20 mL of autoclaved media was poured and allowed to solidify [30].

### 2.3. Extraction of Phytochemicals

Powdered flower material was soaked into methanol in two different containers for 5 days with constant shaking. Filtration of methanolic extract was done and filtrate was acidified up to pH = 3-4 with 0.1 M H_2_SO_4_. Then, extraction was done by using two different solvents: ethanol and chloroform (1:1) [30].

### 2.4. Phytochemical Analysis of Flower Extract

Different phytochemical tests of both chloroform and ethanol flower extract of *H. rosa-sinensis* were carried out by using different reagents like Dragendorff's reagent, NaOH, HCl, and KOH, etc. (Table 1).

#### 2.4.1. Confirmation test for Alkaloids and Betacyanin's

Dragendorff's reagent was used for the determination of alkaloids in flower extract. By adding 1-2 drops of Dragendorff's reagent in both chloroform and ethanol flower extract of *H. rosa-sinensis*. Brick red precipitates formation confirms the presence of alkaloids in flower extract [31]. For the confirmation test of the betacyanin's presence, 2 M NaOH was added in both chloroform and ethanol flower extract of *H. rosa-sinensis*. Yellow precipitates formation confirms the presence of betacyanin's [31].

#### 2.4.2. Confirmation Test for Tannins

Freshly prepared 10% KOH was used for the confirmation of the presence of tannins and glycoside in flower extract. Formation of white precipitates confirm the presence of tannins however, if red precipitates formed it indicates the presence of glycosides [31].

#### 2.4.3. Confirmation Test for Steroids

For the qualitative analysis of steroids in both chloroform and ethanol flower extract, concentrated H_2_SO_4_ was added in flower extract and green precipitates formation confirms the presence of steroids [31].

#### 2.4.4. Confirmation Test for Flavonoids and Quinones

For the confirmation test of the presence of flavonoids and quinones conc. HCl was added in flower extracts, if color changes, it confirms the presence of flavonoids while formation of yellow precipitates confirm presence of quinones [31].

### 2.5. Preparation of Bacterial Inoculum

Inoculum of harmful strain of bacterium was grown in Luria–Bertani (LB) broth. LB broth was made by dissolving 1.25 g of LB in 50 mL of d·H_2_O and pH = 7.0 according to McFarland standard (CFU corresponding to the 0.5 McFarland standard) and finely autoclaved. Four to five colonies of bacterial strain (*E. coli* and foot ulcer bacteria) were inoculated to broth and incubated for 24 h at 37°C [30].

### 2.6. Disc Diffusion Assay

Disc diffusion assay and Agar well diffusion method was done after Bauer et al. [31] to check bactericidal characters of plant removes. Bacterial inoculums (according to 0.5 McFarland standard) were used to make lawn through swabbing. Plates were dried for 10–15 min. Discs were dipped in plant extract (50 mg/mL) and then placed on agar (Each test plate had four discs) [32]. Plates were kept at 37°C for 24 h. After the incubation, plates were examined for zone of inhibition. Inhibition zone was measured using calipers [33].

### 2.7. Antitumor activity

Antitumor potential of plant concentrates of *H. rosa-sinensi*s was determined by using potato disc method as stated by [34].

#### 2.7.1. Preparation of Bacterial Culture

*A. tumefaciens* pathogenic strain (LBA4404) was cultured on LB agar medium. LB medium was set-up by dissolving 1.25 g of LB in 50 mL of refined water and pH was set at 7.0. Media was autoclaved in 100 mL flask. Four to five colonies from culture plate of *A. tumefaciens* were moved to LB stock and brooded for 48 h at 30°C. In a test tube, 10 mL of phosphate (pH 7.2) was taken and around six to seven loops of bacterial suspensions were included in it.

#### 2.7.2. Preparation of Inoculum

Total volume of 1.5 mL of inoculum was prepared from initial stocks by adding 0.15 mL of each of the stock solution in three autoclaved test tubes. Finally, 0.75 mL of double distilled water and 0.6 mL culture of bacteria was added in each test tube. 0.15 mL DMSO replacing flower extracts solution was used as negative control while 0.15 mL of DMSO and 1.35 mL double distilled water was used as blank. So as to maintain a strategic distance from sullying, every arrangement was set-up in laminar stream hood and every single prudent step was considered.

#### 2.7.3. Preparation of Agar Plates

For the preparation of agar plates, 15 gL^−1^ of plane agar was broken up in distilled water and sanitized in an autoclave. In each petri-plate, about 20 mL of autoclaved agar solution was poured and allowed to harden.

#### 2.7.4. Preparation of Potato Discs

Potatoes were washed with tap water, then surface-sanitized by soaking in a 0.1% mercury chloride (HgCl_2_) solution for 10 min. They were then washed three times with distilled water and dried. A disinfected borer was used to cut the potatoes into cylindrical pieces, which were placed in distilled water in petri dishes. Thick discs (5 mm) were cut from the potato cylinders, disinfected with autoclaved water, and transferred to agar plates. A 50 μL inoculum was added to the surface of each disc, allowed to diffuse, and the plates were sealed with parafilm. The sealed plates were then incubated at 28°C for 21 days.

#### 2.7.5. Staining of Potato Discs

Lugol's solution, a mixture of 10% potassium iodide (KI) and 5% iodine in distilled water, was prepared. The potato discs were covered with Lugol's solution and allowed to diffuse for 15 min. The discs were then observed under a light microscope, and the unstained portions were identified as “tumors”. Number of tumors for each disc were calculated. Following formula was used to determine the percentage inhibition of plant extract.

(1) $$\begin{array}{c} \%\text{ inhibition}=100-\frac{\text{No}.\text{ of tumors with extract}}{\text{No}.\text{ of tumors with control}}\times 100. \end{array}$$

### 2.8. Statistical Analysis

ANOVA was done to analyze the data collected by following above protocol by using statistical software SPSS version 25.0.0.

## 3. Results

### 3.1. Phytochemical Analysis of *H. rosa-sinensis*

#### 3.1.1. Phytochemical Analysis of Chloroform Extract of *H. rosa-sinensis*

Phytochemical screening was performed for the qualitative analysis of phytochemicals present in chloroform flower extracts of *H. rosa-sinensis* as shown in Table 1.

**Figure 1:**
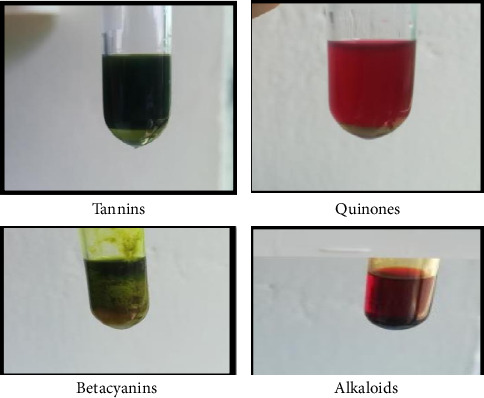
Phytochemical analysis of chloroform extract of *H. rosa-sinensis.*

Phytochemicals were identified and it was found that the compounds present in chloroform extract were alkaloids, betacyanin's, tannins, quinones in nature (Figure 1) while steroids, flavonoids, glycosides were absent.

### 3.2. Phytochemical Analysis of Ethanol Extract of *H. rosa-sinensis*

**Figure 2:**
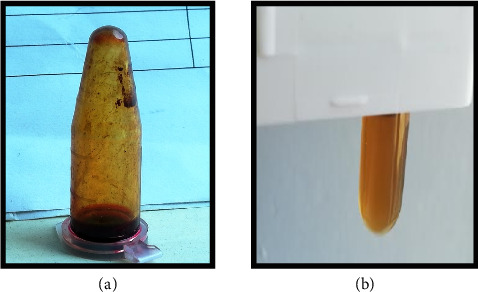
Phytochemical analysis of ethanol extract of *H. rosa-sinensis*. (a) Existence of alkaloids in ethanol extract of *H. rosa-sinensis*. (b) Existence of betacyanin's test in ethanol extract of *H. rosa-sinensis.*

Phytochemical screening was done for the qualitative analysis of phytochemicals present in ethanol flower extracts of *H. rosa-sinensis* as shown in Table 2. Based on biochemical test it was found that the compounds present in ethanol extract were alkaloids, betacyanin's in nature (Figure 2). While steroids, flavonoids, glycosides, tannins, quinones were absent.

### 3.3. Morphological Characterization of Bacteria

The collected bacterial strains were identified through microscopy.

#### 3.3.1. Microscopy

**Figure 3:**
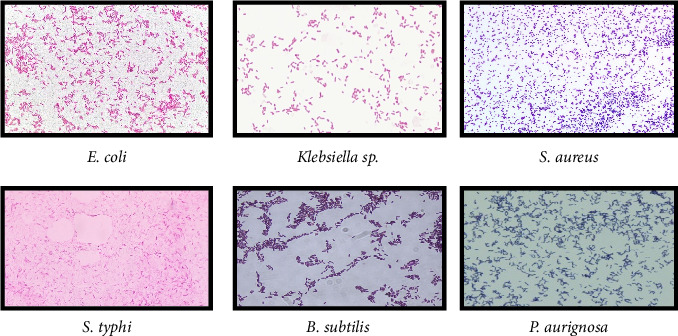
Gram staining of bacteria (scale bar 10 μm at 100 X).

Morphological characteristics of the colonies of bacteria were observed. Bacteria were subjected to gram staining. Our results showed that required strains were gram positive and gram-negative bacteria having rod and round colonies (Figure 3).

### 3.4. Antimicrobial Activity

The in vitro antimicrobial activity of chloroform flower extract and ethanol flower extract of *H*. *rosa-sinensis* was tested on gram negative bacterium; *E. coli, Klebsiella* sp.*, P*. *aeruginosa* and on gram positive bacterium; *S*. *aureus, B*. *subtilis* and *S*. *typhi* isolated from foot ulcers of diabetic patient by using disc diffusion assay.

### 3.5. Chloroform Flower Extract of *H. rosa-sinensis*

**Figure 4:**
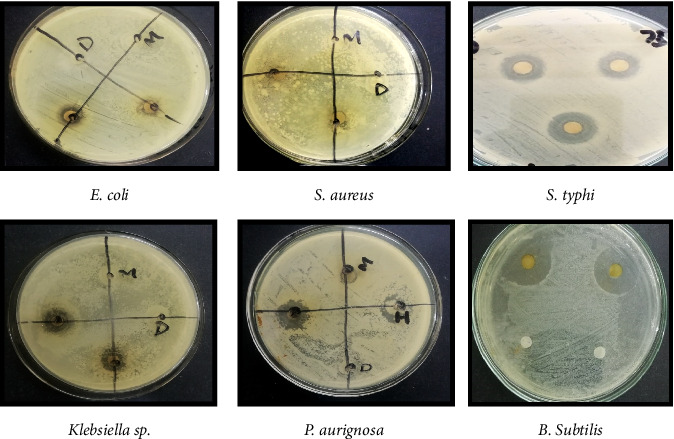
Zones of inhibition observed against bacteria by using chloroform flower extract of *H. rosa-sinensis*.

The diameter of zones of inhibition of chloroform flower extract against *E. coli, S*. *aureus, Klebsiella* sp.*, P*. *aeruginosa*, and *B*.*subtilis, S*. *typhi* was 11, 8, 13, 17, 10, and 23 mm, respectively (Figure 4, Table 3).

#### 3.5.1. Ethanol Flower Extract of *H*. *rosa-sinensis*

**Figure 5:**
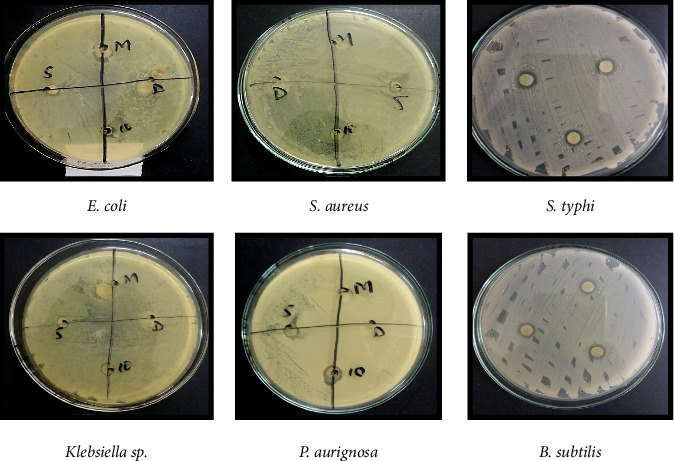
Zones of inhibition observed against bacteria by using ethanol flower extract of *H. rosa-sinensis*.

After 24 h zones of inhibition were observed and measured using ruler. The diameter of zones of inhibition of ethanol flower extract against *E. coli, S*. *aureus, Klebsiella* sp., foot ulcer bacteria of diabetic patient and *B*. *subtilis, S*. *typhi* were 6, 0,6, 10, 8, and 10 mm, respectively (Figure 5, Table 3).

#### 3.5.2. Overall Antibacterial Activity

It was observed that chloroform extract of *Hibiscus rosa-sinensis* showed significant (*p* ≤ 0.05) antibacterial activity against *S*. *typhi* than *E. coli, S*. *aureus, Klebsiella* sp.*, P*. *aeruginosa* and *B*. *subtilis.* While the ethanolic extract of *Hibiscus* has more significant antibacterial activity against foot ulcer bacteria of diabetic patient than *E. coli, Klebsiella* sp.*, P*. *aeruginosa* and *B*. *subtilis.* But the ethanol extract showed no antibacterial activity against *S*. *aureus*.

### 3.6. Antitumor Activity

The antitumor activity of chloroform and ethanol flower extract of *H*. *rosa-sinensis* Linn was tested by potato disc antitumor bioassay by using *A tumifaciens*. Flower extract inhibited the development of crown gall tumors on discs in solvent dependent manner.

#### 3.6.1. Chloroform Flower Extract of *H*. *rosa-sinensis*

**Figure 6:**
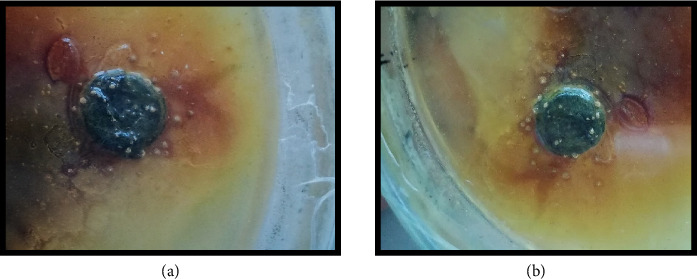
(a) No. of tumors with control (b) tumor inhibition by chloroform flower extract of *H*. *rosa-sinensis.*

After the incubation period of 21 days the potato discs were analyzed. The chloroform flower extract of *H*. *rosa-sinensis* linn produced antitumor effect with 30.5% inhibition of tumor formation (Figure 6).

#### 3.6.2. Ethanol Flower Extract of *H*. *rosa-sinensis*

**Figure 7:**
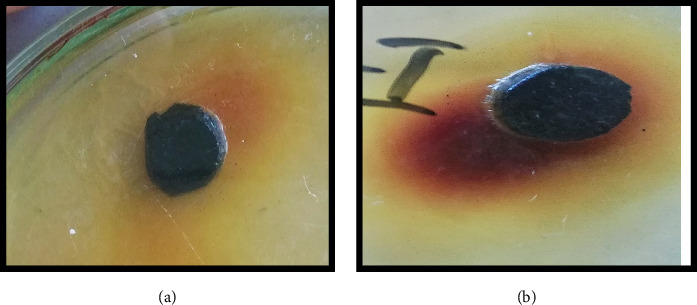
(a) No. of tumors with control (b) tumor inhibition by ethanol flower extract of *Hibiscus rosa-sinensis*.

The ethanol flower extract of *H*. *rosa-sinensis* produced strong antitumor effect with 95.7% inhibition of tumor formation (Figure 7). It was observed that chloroform extract of *H*. *rosa-sinensis* showed significant (*p* ≤ 0.05) antitumor activity by inhibiting the growth of crown gall tumors. While the ethanolic extract of *H*. *rosa-sinensis* has more significant antitumor activity as it inhibited the formation of crown gall tumors with large percentage of inhibition write some more description.

## 4. Discussion

*Hibiscus rosa-sinensis* has been extensively studied for its phytochemical composition and medicinal properties. The quality and quantity of extracts in plant shows the power of pharmacological and natural impact of that specific plant. Initial phase in phytochemical analysis is to remove the dynamic plant segments from the part by applying a suitable extraction technique and picking an appropriate solvent for extraction. The total phytochemical in a plant differ comparative to dry weight of plant material and relies upon the geographical zone, part of the plant utilized and the development stage [35]. Phytochemical screening of various plant parts revealed the presence of alkaloids, glycosides, flavonoids, tannins, saponins, and terpenoids [36–38]. In this study it was found that chloroform extract of *H*. *rosa-sinensis* contains alkaloids, betacyanin's, tannins, and quinones in nature while in ethanol extract only alkaloids and betacyanin's were found.

The in vitro antimicrobial activity of chloroform flower extract and ethanol flower extract of *Hibiscus rosa-sinensis* was tested on *E. coli, K*. *pneumoniae, S*. *aureus, B*. *subtilis, Salmonella typhi* and *P*. *aeruginosa* isolated from foot ulcer of diabetic patient by using disc diffusion assay. Previous studies reported that this plant exhibits significant antibacterial activity against various pathogens, including *E*. *coli*, *P*. *aeruginosa*, and *S*. *aureus* [37, 38].

Foot infections in diabetic patients are mostly polymicrobial due to the involvement of aerobic and anaerobic bacteria. *Streptococcus* spp., *Enterobacteriaceae* and *Staphylococcus* spp., are aerobic microbial agents while anaerobic bacteria include *Bacteroides* spp., *Clostridium* sp. [39, 40]. *B. subtilis* incorporate bacteremia, endocarditis, pneumonia, and septicemia. However, these diseases were found in immuno-compromised patients. *Salmonella typhi,* typhoid fever is brought about by *Salmonella* serotypes. It damages organs like kidney, liver and spleen. In extreme types of the sickness, enough fluid and electrolytes are lost to disturb the water-salt digestion, decline the flowing blood volume and blood vessel weight, and cause hypovolemic shock. Oliguria and azotemia may create in serious cases in result of renal association because of hypoxia and toxemia.

The diameter of zones of inhibition of chloroform flower extract against *E. coli, S*. *aureus, Klebsiella* sp., foot ulcer bacteria of diabetic patient and *Bacillus subtilis, S*. *typhi* was 11, 8, 13, 17, 10, and 23 mm, respectively. While the ethanolic extract of *H*. *rosa-sinensis* linn has more significant antibacterial activity against foot ulcer bacteria of diabetic patient than *E. coli, Klebsiella* sp., foot ulcer bacteria of diabetic patient and *B*. *subtilis.* But the ethanol extract showed no antibacterial activity against *S*. *aureus.*

It was observed that chloroform extract of *H. rosa-sinensis* showed significant antibacterial activity against *S. typhi* than *E. coli, S. aureus, Klebsiella* sp., foot ulcer bacteria of diabetic patient and *Bacillus subtilis.* While the ethanolic extract of *H. rosa-sinensis* has more significant antibacterial activity against foot ulcer bacteria of diabetic patient than *E. coli, Klebsiella* sp., foot ulcer bacteria of diabetic patient and *Bacillus subtilis.* But the ethanol extract showed no antibacterial activity against *S. aureus.*

The antitumor activity of chloroform and ethanol flower extract of *H*. *rosa-sinensis* was tested by potato disc antitumor bioassay by using *A. tumifaciens*. A gram negative bacterium, *A. tumifaciens,* that causes tumor in various plant species [41]. It is a neoplastic illness caused by Ti-plasmid present in this bacterium. Ti-plasmid contains the hereditary data known as T-DNA which stimulates self-directed tumors in plant by following disintegration [42]. T-DNA induces proliferation cells in plants without prompting apoptosis stimulates tumor induction just like cancer development in animals and humans [43, 44]. *Helicobacter pylori* [45] and *Bartonella henselae* [46], these bacteria that cause tumor development in man, have same pathogenicity technique as followed by *A. tumefaciens* [47, 48]. Plant extracts are playing an essential role in development of new antitumor drugs like especially alkaloids. Alkaloids inhibit topo-isomerase, which is directly involved in DNA replication, causing apoptosis [49, 50]. Betacyanins are also helpful in preventing cancer and cardiovascular diseases because of its antioxidant property. A lot of plant products like vinblastine, and vincristine derivatives of podophyllotoxin including etoposide and camptothecin have been used against tumor [50].

Flower extract inhibited the development of crown gall tumors on discs in solvent dependent manner. The chloroform flower extract of *Hibiscus rosa-sinensis* produced antitumor effect with 30.5% inhibition of tumor formation. While the ethanol flower extract of *Hibiscus rosa-sinensis* linn produced strong antitumor effect with 95.7% inhibition of tumor formation. Likewise *Hibiscus manihot* L. flower extract (HML) has been reported to suppressed the proliferation of A549 cells [51]. Overall, we can say that the ethanolic extract of *Hibiscus rosa-sinensis* has more significant antitumor activity than chloroform extract as it inhibited the formation of crown gall tumors with large percentage of inhibition.

## 5. Conclusion

Hence, the result of present study indicated that the both chloroform and ethanol extracts of *H*. *rosa-sinensis* showed significant antibacterial and antitumor potential. But in the case of antibacterial property chloroform extract has shown more resistance as compared to ethanol extract. While in the case of antitumor property ethanol extract has more potential to inhibit tumor formation as compared to chloroform extract.

## Figures and Tables

**Table 1:** Phytochemical analyses of chloroform extract *H. rosa.sinensis*.

Phytochemicals	Reagent	Precipitates	Inference
Alkaloids	Dragendorff's reagent	Brick red ppts formed.	Alkaloids are present.
Betacyanin's	2 M NaOH	Yellow ppts formed.	Betacyanin's are present.
Tannins	10% KOH	White ppts formed.	Tannins are present.
Glycoside	10% KOH	No red ppts formed.	Glycosides are absent.
Steroids	Conc. H_2_SO_4_	No green ppts formed.	Steroids are absent.
Flavonoids	Conc. HCl	No color changes.	Flavonoids are absent.
Quinones	Conc. HCl	Yellow ppts formed.	Quinones are present.

*Note:* ppts = precipitates.

**Table 2:** Phytochemical analysis of ethanol extract of *H. rosa-sinensis*.

Phytochemicals	Reagent	Precipitates	Inference
Alkaloids	Dragendorff's reagent	Brick red ppts formed.	Alkaloids are present.
Betacyanin's	2 M NaOH	Yellow ppts formed.	Betacyanin's are present.
Tannins	10% KOH	No white ppts formed.	Tannins are absent.
Glycoside	10% KOH	No red ppts formed.	Glycosides are absent.
Sterols	Conc. H_2_SO_4_	No green ppts formed.	Sterols are absent.
Flavonoids	Conc. HCl	No color changes.	Flavonoids are absent.
Quinones	Conc. HCl	No yellow ppts formed.	Quinones are absent.

*Note:* ppts mean precipitates.

**Table 3:** Zones of inhibition of *H. rosa-sinensis* flower extract against bacterial strains.

Bacterial strains	Diameter of zones of inhibition (mm)			
	Distilled water	Streptomycin (100 μg/disc)	Chloroform extract	Ethanol extract
*E. coli*	0 mm	15 ± 0.05	^∗^11 ± 0.05^c^	6 ± 0.03^c^
*S. aureus*	0 mm	15 ± 0.05	8 ± 0.02^d^	0
*Klebsiella sp.*	0 mm	15 ± 0.05	13 ± 0.01^c^	6 ± 0.02^c^
*P. aurignosa*	0 mm	15 ± 0.05	17 ± 0.05^b^	10 ± 0.03^a^
*B. subtilius*	0 mm	15 ± 0.05	12 ± 0.01^c^	8 ± 0.02^b^
*S. typhi*	0 mm	15 ± 0.05	23 ± 0.01^a^	10 ± 0.02^a^

*Note:* Different alphbetical letter indicate the significant diference.

## Data Availability

The data that support the findings of this study are available from the corresponding author upon reasonable request.
